# Exploring the Latest Advances in Public Health and Epidemiology Informatics

**DOI:** 10.1055/s-0044-1800754

**Published:** 2025-04-08

**Authors:** Gayo Diallo, Georgeta Bordea, Cécilia Samieri

**Affiliations:** 1 Team AHeaD - Inserm Bordeaux Population Health Research Center, Univ. Bordeaux, 146 rue Léo Saignat, F-33000 Bordeaux, France; 2 Univ. La Rochelle, L3i, EA 2118, F-17000, La Rochelle, France

**Keywords:** Public Health, Epidemiology Informatics, Precision Prevention, IMIA Yearbook 2024

## Abstract

**Objectives** : The objective of this review was to identify and analyze the most recent research and prevailing trends in the field of Public Health and Epidemiology Informatics (PHEI).

**Methods** : We adopted a methodical search approach that was similar to the one used in the previous edition of the PHEI section's synopsis. We conducted a thorough search on PubMed using an extensive range of keywords that cover topics related to public health, epidemiological surveillance, and medical informatics. As a result, there were 840 publications found on PHEI. The three section editors carefully examined the references. Afterwards, nine articles were selected as potential contenders for the “best paper” awards. The candidates underwent a thorough peer-review process that included six external reviewers, as well as the section editors and the two chief editors of the IMIA Yearbook of Medical Informatics. Every paper was subjected to a total of five reviews.

**Results** : The search yielded 840 references, and after review of the nine “best paper” candidates, only two papers emerged as strong contenders for the “best paper” award. The first candidate paper, which received a broader consensus, explored the integration of clinical language models in medicine. This model envisioned working alongside physicians, providing real-time guidance at the point of care. The second candidate fo-cused on developing personalized digital interventions to effectively increase short-term physical activity.

**Conclusion** : The recent PHEI section review has identified a significant rise in the quantity of pertinent stud-ies in comparison to the previous edition. The search strategy for this year incorporated precision medicine-related keywords for the first time, which may have led to an increased number of retrieved publications specifically related to PHEI.

## 1. Introduction

Public Health and Epidemiology Informatics (PHEI) continues to evolve rapidly, playing a critical role in transforming population health through the strategic application of information technology. While traditionally PHEI has focused on large datasets for population-level analysis, a growing trend in 2023 is the integration of precision medicine principles [ 7 ]– tailoring interventions and preventative measures to individual risk factors. This personalized approach to public health, often referred to as “Precision Prevention”, holds immense potential for improving health outcomes and optimizing resource allocation.

This synopsis paper delves into the latest research published in 2023 that leverages PHEI tools and methodologies to advance precision prevention strategies. We explore the innovative ways researchers use digital technologies and data analysis to identify individuals at high risk for specific health conditions, allowing for targeted interventions and preventive measures. We examine the best published papers in this field, highlighting the potential and challenges associated with integrating personalized approaches into public health initiatives.

Following the previous edition of the IMIA Yearbook focused on One health [ 6 ], the present synthesis in the field of PHEI examines the scientific literature that emerged in 2023 in the field of medical informatics, with a focus on the subfield of public health and epidemiology. This analysis, unlike previous years, seeks to identify novel topics and trends in this field. In addition, it describes the detailed procedure used to select the best articles published in 2023. The process includes the section editors who supervise the peer review of the most representative research papers chosen for their relevance, excellence and originality.

## 2. Methods

Similarly to the last edition of the IMIA Yearbook for the PHEI section [ 2 ], a comprehensive literature search was performed by the section editors using the PubMed/MEDLINE database from the National Center for Biotechnology Information (NCBI). A large set of MeSH descriptors were used to retrieve relevant studies ranging from January 1, 2023 to December 31, 2023. The queries targeted public health ( *e.g.* , “Public health”, “Public health practice”, “Registries”, “Population surveillance”) or epidemiological journal articles ( *e.g.* , “Epidemiological Monitoring”, “Epidemiologic surveillance”, “Disease Outbreak”), which included medical informatics topics ( *e.g.* , “Medical informatics”, “Health Information Systems”, “Neural networks”). This year, a new set of keywords related to precision medicine was added: “Precision Medicine”, “Personalized Medicine”. Returned references addressing topics that could be addressed by other sections of the Yearbook were excluded. These references include in particular COVID-19 (including MeSH terms “COVID-19”, “Pandemics”, “SARS-CoV-2”).

**Figure 1.**
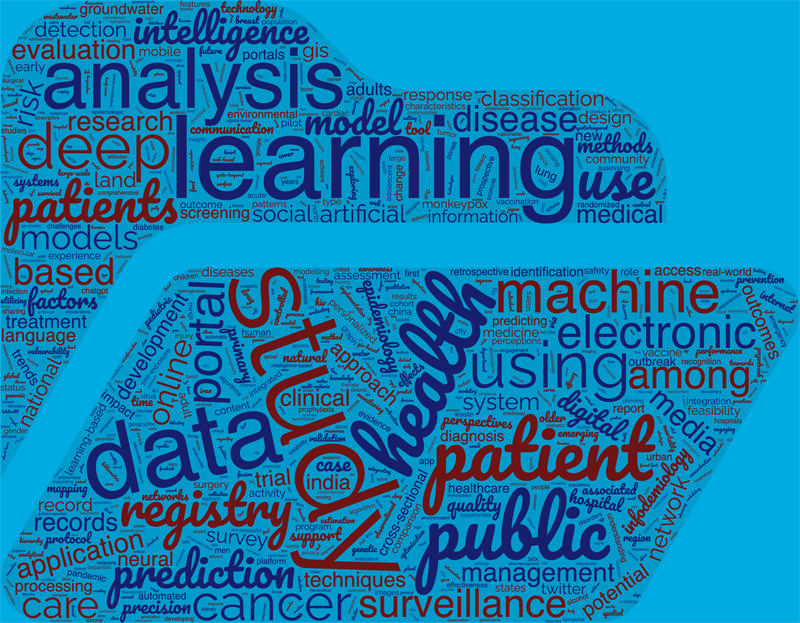
Word cloud of terms which appeared in the title of the 840 retrieved papers

The study was performed in early February 2024, and the search returned a total of 840 references, which was greater than for the 2022 Yearbook edition (where 534 references were retrieved). Figure 1 depicts a cloud of the main terms which appear in the title of these articles. Unsurprisingly, we can find terms such as “online portal”, “deep learning”, “surveillance” and “data”.

The 840 articles were reviewed separately by the three section editors, considering the number of citations for each reference in the process. They were first classified using the BibReview tool [ 10 ] into three categories: keep, discard, or pending. Then, the three section editors jointly reviewed these references and selected a consensual list of nine candidate best papers. These papers are listed in Table 1 following ascendant order by PMID.

**Table 1.** List of the nine candidate papers for the PHEI section.

#	PMID	Authors & Ref.	Title
1	36644659	Lee et al., 2023	Internet of medical things-based real-time digital health service for precision medicine: Empirical studies using MEDBIZ platform
2	36684850	Liu et al., 2023	Clarifying sleep characteristics and analyzing risk factors of sleep disorders to promote a predictive, preventive, and personalized medicine in patients with burn scars
3	36698426	Hall et al., 2023	‘Putting all my eggs into the app’: Self, relational and systemic surveillance of mothers' use of digital technologies during the transition to parenting
4	36845417	Gómez-Carrillo et al., 2023	Restoring the missing person to personalized medicine and precision psychiatry
5	36869927	Cascella et al., 2023	Evaluating the feasibility of ChatGPT in healthcare: an analysis of multiple clinical and research scenarios
6	37286606	Jiang et al., 2023	Health system-scale language models are all-purpose prediction engines
7	37649068	OKeeffe et al., 2023	Strengthening community-based surveillance: lessons learned from the 2018-2020 Democratic Republic of Congo (DRC) Ebola outbreak
8	37794870	Javed et al., 2023	Personalized digital behaviour interventions increase short-term physical activity: a randomized control crossover trial substudy of the MyHeart Counts Cardiovascular Health Study
9	38264687	Fu et al., 2023	Early monitoring-to-warning Internet of Things system for emerging infectious diseases via networking of light-triggered point-of-care testing devices

The full text of all of these nine papers was then peer-reviewed by the three PHEI section editors, one IMIA Yearbook chief editor and five external reviewers. Each paper was reviewed by at least five reviewers to ensure an informed decision making.

## 3. Results and Discussion

### 3.1. Overall observation

This year we observed an increase in the number of papers published in 2023 compared with 2022. Extending the keywords to precision and personalized medicine could have impacted the obtained results.

Of the 840 papers retrieved from PubMed, nine articles were selected for a detailed evaluation. These nine articles focused mainly on the following issues: (i) the use of large language models and natural language processing techniques [ 1 , 7 ] to be used in clinical settings, such as complementing physicians' work and providing guidance at the point of care as addressed by Jiang *et al.* [ 7 ]; (ii) personalized and precision computational approaches to improve efficiency of digital interventions, in particular applied to cardiovascular [ 8 ], psychiatric [ 4 ] and sleep disorder domains [ 12 ]; (iii) Internet of Things-based approaches to address health issues [ 3 , 11 ]; and finally (iv) digital tools-based surveillance, addressing in particular Ebola outbreak [ 13 ] and the transition of mothers to parenting [ 5 ]. As in previous years, we noted a preponderance of machine learning approaches to address different PHEI questions.

### 3.2. Selected papers

Among the nine candidate papers suggested for this year's reviewing process, two best papers were selected for presentation during the final consensus meeting [ 7 , 8 ]. We considered the study by Jiang *et al.* [ 7 ] as very aligned with the special topic of this year's IMIA Yearbook. It describes a study which leverages natural language processing techniques for large language models that are trained and tuned on medical language. It shows that using clinical language models in medicine could help read alongside physicians and provide guidance at the point of care. The second best paper, by Javed *et al.* [ 8 ], describes how tailored digital interventions could be effective in increasing short-term physical activity in a free-living cohort. A detailed summary of these two papers is provided in the Appendix.

## 4. Conclusion

The 2023 selection process of PHEI confirmed the trend towards addressing public health and computational epidemiology issues with machine learning and deep learning techniques. After a decrease in the number of papers published on PHEI in 2022, this year's figures have returned to the levels usually observed. Using additional keywords related to precision and personalized medicine may have contributed to this result.

## References

[1] Cascella M, Montomoli J, Bellini V, Bignami E. Evaluating the Feasibility of ChatGPT in Healthcare: An Analysis of Multiple Clinical and Research Scenarios. J Med Syst. 2023 Mar 4;47(1):33. doi: 10.1007/s10916-023-01925-4 10.1007/s10916-023-01925-4 PMC9985086 36869927

[2] Diallo G, Bordea G, Samieri C. Section Editors for the IMIA Yearbook Section on Public Health and Epide-miology Informatics. Broad Trends in Public Health and Epidemiology Informatics. Yearb Med Inform. 2023 Aug;32(1):264-268. doi: 10.1055/s-0043-1768754. 10.1055/s-0043-1768754 PMC10751154 38147868

[3] Fu Y, Liu Y, Song W, Yang D, Wu W, Lin J, et al. Early monitoring-to-warning Internet of Things system for emerging infectious diseases via networking of light-triggered point-of-care testing devices. Exploration (Beijing). 2023 Oct 5;3(6):20230028. doi: 10.1002/EXP.20230028 10.1002/EXP.20230028 PMC10742204 38264687

[4] Gómez-Carrillo A, Paquin V, Dumas G, Kirmayer LJ. Restoring the missing person to personalized medicine and precision psychiatry. Front Neurosci. 2023 Feb 9;17:1041433. doi: 10.3389/fnins.2023.1041433 10.3389/fnins.2023.1041433 PMC9947537 36845417

[5] Hall J, Hiebert B, Facca D, Donelle L. 'Putting all my eggs into the app': Self, relational and systemic surveillance of mothers' use of digital technologies during the transition to parenting. Digit Health. 2023 Jan 18;9:20552076221150742. doi: 10.1177/20552076221150742 10.1177/20552076221150742 PMC9869190 36698426

[6] Hollis KF, Mougin F, Soualmia LF. Informatics for One Health. Yearb Med Inform. 2023 Aug;32(1):2-6. doi: 10.1055/s-0043-1768757. 10.1055/s-0043-1768757 PMC10994713 38575142

[7] Jiang, LY, Liu, XC, Nejatian, NP, Nasir-Moin M, Wang D, Abidin A, et al. Health system-scale language models are all-purpose prediction engines. Nature. 2023 Jul;619:357–362. doi: 10.1038/s41586-023-06160-y 10.1038/s41586-023-06160-y PMC10338337 37286606

[8] Javed A, Kim DS, Hershman SG, Shcherbina A, Johnson A, Tolas A, et al. Personalized digital behaviour in-terventions increase short-term physical activity: a randomized control crossover trial substudy of the MyHeart Counts Cardiovascular Health Study. Eur Heart J Digit Health. 2023 Aug 9;4(5):411-419. doi: 10.1093/ehjdh/ztad047 10.1093/ehjdh/ztad047 PMC10545510 37794870

[9] König I, Fuchs O, Hansen G, von Mutius, E, Kopp MV. What is precision medicine. Eur Respir J. 2017 Oct 19;50(4):1700391. doi:10.1183/13993003.00391-2017 10.1183/13993003.00391-2017 29051268

[10] Lamy JB, Séroussi B, Griffon N, Kerdelhué G, Jaulent MC, Bouaud J. Toward a formalization of the process to select IMIA Yearbook best papers. Methods Inf Med, 2015;54(2):135–144. doi: 10.3414/ME14-01-0031 10.3414/ME14-01-0031 25396220

[11] Lee HY, Lee KH, Lee KH, Erdenbayar U, Hwang S, Lee EY, et al. Internet of medical things-based real-time digital health service for precision medicine: Empirical studies using MEDBIZ platform. Digit Health. 2023 Jan 9;9:20552076221149659. doi: 10.1177/20552076221149659 10.1177/20552076221149659 PMC9834931 36644659

[12] Liu H, Shu F, Ji C, Xu H, Zhou Z, Wang Y, et al. Clarifying sleep characteristics and analyzing risk factors of sleep disorders to promote a predictive, preventive, and personalized medicine in patients with burn scars. EPMA J. 2023 Jan 9;14(1):131-142. doi: 10.1007/s13167-022-00309-x 10.1007/s13167-022-00309-x PMC9838372 36684850

[13] OKeeffe J, Takahashi E, Otshudiema JO, Malembi E, Ndaliko C, Munihire NM, et al. Strengthening community-based surveillance: lessons learned from the 2018–2020 Democratic Republic of Congo (DRC) Ebola outbreak. Confl Health 2023 Aug 30;17(1):41. doi: 10.1186/s13031-023-00536-7 10.1186/s13031-023-00536-7 PMC10466702 37649068

